# Pathophysiology of sepsis‐induced cholestasis: A review

**DOI:** 10.1002/jgh3.12771

**Published:** 2022-05-25

**Authors:** Maria Iuliana Ghenu, Dorin Dragoş, Maria Mirabela Manea, Dorin Ionescu, Lucian Negreanu

**Affiliations:** ^1^ 1st Department Medical Semiology (MIG, DD, DI), 6th Department Clinical Neurosciences (MMM), 5th Department Internal Medicine (LN) “Carol Davila” University of Medicine and Pharmacy Bucharest Romania; ^2^ 1st Internal Medicine Clinic University Emergency Hospital Bucharest Romania; ^3^ Neurology Department National Institute of Neurology and Cerebrovascular Diseases Bucharest Romania; ^4^ Nephrology Clinic University Emergency Hospital Bucharest Romania; ^5^ Gastroenterology Clinic University Emergency Hospital Bucharest Romania

**Keywords:** bile acids, carrier proteins, hepatocytes, inflammation, lipopolysaccharide

## Abstract

Sepsis is a critical condition resulting from the excessive activation of the inflammatory/immune system in response to an infection, with high mortality if treatment is not administered promptly. One of the many possible complications of sepsis is liver dysfunction with consequent cholestasis. The aim of this paper is to review the main mechanisms involved in the development of cholestasis in sepsis. Cholestasis in a septic patient must raise the suspicion that it is the consequence of the septic condition and limit the laborious attempts of finding a hepatic or biliary disease. Prompt antibiotic administration when sepsis is suspected is essential and may improve liver enzymes. Cholestasis is a syndrome with a variety of etiologies, among which sepsis is frequently overlooked, despite a number of studies and case reports in the literature demonstrating not only the association between sepsis and cholestasis but also the role of cholestasis as a prognostic factor for sepsis‐induced death.

## Graphical abstract

The aim of this paper is to review the main mechanisms involved in the development of cholestasis in sepsis. Cholestasis is a syndrome with a variety of etiologies, among which sepsis is frequently overlooked, despite a number of studies and case reports in the literature demonstrating not only the association between sepsis and cholestasis but also the role of cholestasis as a prognostic factor for sepsis‐induced death.

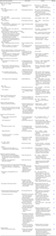

## Introduction

Sepsis is a severe, life‐threatening complication of an infection. It occurs when the immune response becomes inadequate, triggering organ dysfunction. Sepsis involves a pro‐inflammatory and antiinflammatory response leading to organ failure.^1^ Sepsis may progress to septic shock, a condition associated with high mortality, defined as sepsis leading to metabolic and hemodynamic dysfunction severe enough to push lactacidemia above 2 mmol/L and to pull mean blood pressure below 65 mmHg, unresponsive to appropriate fluid resuscitation, and requiring vasopressor support.^1^ Cholestasis occurs in up to 40% of critical patients, sepsis being one of its multiple causes.^2^ According to Surviving Sepsis Campaign Guidelines, liver dysfunction associated with sepsis is defined by a rise in serum bilirubin above 2 mg/dL accompanied by defective coagulation reflected by an international normalized ratio (INR) above 1.5.^3^ Sepsis‐associated liver dysfunction includes hypoxic hepatitis, sepsis‐induced cholestasis, and faulty protein synthesis generating coagulopathy.^3^ Hypoxic hepatitis is defined by (i) clinical signs of cardio‐circulatory and/or respiratory failure, (ii) a sudden and transient rise in serum transaminases (>20 times the upper limit of normal), and (iii) absence of other causes of hepatic necrosis.^4^ Hypoxic hepatitis may be associated with prolonged prothrombin time, acute kidney injury, and higher bilirubin.^4^ Notwithstanding preserved cardiac output, septic shock generates a craving for oxygen unmet by oxygen delivery and extraction, compounded by altered hepatic perfusion due to vasodilation–vasoconstriction imbalance.^4^ Endotoxins and inflammatory mediators too may promote hypoxic hepatitis.^4^ Sepsis‐induced cholestasis results from: (i) impairment of bile acids and bilirubin uptake and transport due to hypoxia and hypoperfusion; (ii) deleterious effects of endotoxins and inflammatory cytokines on the genic expression of the proteins transporting bile acids and bilirubin, on the cytoskeleton architecture around the bile ducts, and on the tight junctions between hepatocytes.^4^

**Figure 1:**
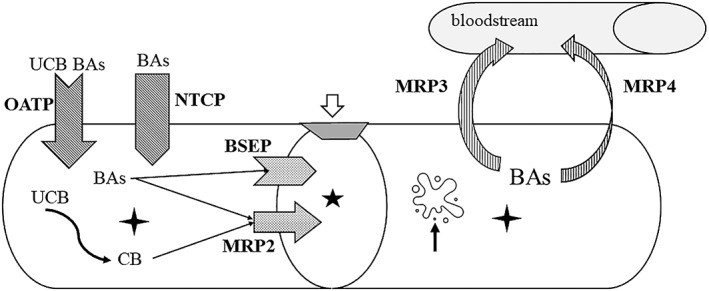
Sepsis‐induced disruption of the: (i) Cytoskeletal architecture (black arrow) in the liver cells (four points star) lining the bile canaliculi (five points star), (ii) tight junctions (open arrow) between hepatocytes, and (iii) transporter proteins' activity in liver cell membrane: sodium‐taurocholate co‐transporting polypeptide (NTCP) (which transports bile acids from plasma into hepatocytes), organic anions transporting polypeptides (OATP) (which transports conjugated and unconjugated bile acids and unconjugated bilirubin into hepatocytes), bile salt export pump (BSEP) (which transports bile salts into bile ducts), multidrug resistance protein (MRP) 2 (which transports conjugated bile acids and conjugated bilirubin from hepatocytes into bile ducts). MRP 3 and MRP 4 mediate the expulsion of bile acids from hepatocyte into the bloodstream, acting as a protective mechanism activated in cholestatic conditions. BAs, bile acids; BSEP, bile salt export pump; CB, conjugated bilirubin; UCB, unconjugated bilirubin.

The main hepatocellular transport proteins involved in sepsis‐induced cholestasis are listed in Table 1 and their action mechanism is summarized in Figure 1. Sepsis‐induced liver dysfunction is associated with high mortality; prompt infection control is a key step in improving the prognostic.^4^

**Table 1:** The main hepatocellular transport proteins and their principal functions

Hepatocellular transport proteins	Function	Reference
Sodium‐taurocholate co‐transporting polypeptide	Bile acids transport from plasma into the hepatocyte	5
Organic anion‐transporting polypeptides	Uptake of many compounds such as conjugated and unconjugated bile acids and unconjugated bilirubin into the hepatocytes	6, 7
Bile salt export pump	Bile salts excretion into the bile ducts	8
Multidrug resistance‐associated protein 2	Conjugated bile acids excretion from hepatocytes into bile	9
	Transport non‐bile salt organic anions, reduced glutathione, and mono‐ and bisglucuronosyl bilirubin from hepatocytes into the bile ducts	7, 10
Multidrug resistance‐associated proteins 3 and 4	Expulsion of bile acids into the bloodstream	9

## Methods

A PubMed (https://www.ncbi.nlm.nih.gov/pubmed) search for “cholestasis[Title/Abstract] AND sepsis[Title/Abstract]” provided the articles employed in this review. The authors endeavored to include (almost) all the relevant papers, giving priority to those attempting to define the pathophysiology of sepsis‐associated liver dysfunction. The results of this search are summarized in Table 2.

**Table 2:** The main mechanisms involved in the development of sepsis‐associated cholestasis and reports of sepsis‐related cholestasis (H, human study; T, in vitro study; V, in vivo study)

Sepsis model (where applicable)	Type of study	Findings	Reference
Mechanisms interfering with membrane pumps activity			
NTCP + Na + K + ‐ATPase			
LPS i.p.	(V) Sprague–Dawley rats or C57BL/6 mice	TNF‐α and IL 1β → ↓ NTCP (mRNA, expression and uptake); ↓ Na + K + ‐ATPase → cholestasis and inflammation	13
NTCP + MRP			
LPS (1 mg/kg body weight i.p.) +/− ICKT for 4 weeks	(V) Male Sprague–Dawley rats	LPS: ↓ bile flow, ↓ biliary bile salts and glutathione excretion, ↓NTCP and MRP 2	16
		ICKT: less intense ↓ bile flow, normalization of glutathione excretion, ↑ MRP 2 → partially prevents LPS‐induced cholestasis	16
NTCP + MRP+ BSEP			
Autologous feces i.p +/− adequate resuscitation	(V) Domestic female pigs	↓ NTCP and BSEP, ↑ MRP 4, ↓ MRP 2	15
BSEP + MRP			
LPS (2.5 mg/kg i.v.)	(V) Male Sprague–Dawley rats	↓ BSEP, ↓ MRP 2 → ↓ biliary excretion, ↓ bile flow	14
NTCP + ecto‐ATPase			
*Escherichia coli* LPS (0.1 mg/100 g body i.p. daily for 3 days) TNF‐α (100 μg/kg body i.p.)	(V) Adult male Sprague–Dawley rats	↓ NTCP and canalicular ecto‐ATPase → ↓taurocholate transport → sepsis‐associated cholestasis	20
Rlst‐1 + NTCP			
Bile duct ligation model or cecum ligation and puncture model	(V) Male Sprague–Dawley rats	↓ rlst‐1 mRNA → ↓ taurocholate Transport (sodium‐independent manner)	21
↓ Bile acid secretion into bile and ↓ organic anion transport			
Sepsis model: LPS i.p., SIRS model: sterile abscess formation (turpentine i.m.); bile acids (cholyltaurine and chemodeoxycholyltaurine) and organic anion (Sulfolithocholyltaurine)	(V) Rats	Sepsis: ↓ transport of bile acids and organic anions SIRS: no alteration of transport	26
LPS 0.3 mg/100 g body i.p.; bile acids (cholyltaurine and chenodeoxycholyltaurine) and organic anions (sulfobromophthalein and sulfolithocholyltaurine)	(V) Male Sprague–Dawley rats	↓ Basolateral and canalicular bile acid ↓ organic anion transport, ↓ ATP‐stimulated transport → cholestasis	27
↓ Bile acids excretion			
LPS +/− SEB (50 μg/kg) infused into the IVC or IPV	(V) Adult male Sprague–Dawley rats	↓ Bile acid excretion	28
Endotoxin (7.5 mg/kg i.v.) or monoclonal anti‐TNF‐α antibody followed by endotoxin	(V, T) Male Sprague–Dawley rats, rat hepatocytes	↓ Basal bile flow and salt excretion, ↓ bile salt stimulated bile flow; anti‐TNF‐α antibody blocked endotoxin‐associated cholestasis	29
↓ Bile salts uptake by hepatocyte, ↓ bile acids secretion into bile			
Endotoxin production by eight common bacterial pathogens	(T) Hepatocytes from male Sprague–Dawley rat livers	↓ Bile salt uptake	30
Human stool suspension (1.2 μL/g body weight i.p) *Candida albicans* (2.5 × 10^4^ CFU/g body weight i.v. or 5 × 10^7^ CFU/animal i.p).	(V) Male, 17–20‐week‐old C57BL/6 mice	*C. albicans* infection ↑ conjugated bile acids, ↓ hepatic uptake; PCI: ↑ unconjugated bile acids, defects in secretion	31
Mechanisms interfering with nuclear receptors			
FXR			
LPS (20 mg/kg for 6 h; 30 mg/kg for survival assays, i.p.)	(V, T) Specific pathogen‐free male C57BL/6 mice Human acute monocytic leukemia cell line THP‐1	Endotoxemia: ↑ OSTβ,↓ NTCP and BSEP → ↑ bile acids, ↓ FXR → ↑ NLRP3 inflammasome activation	35
Extensive surgery (“surgical critical illness”) or extensive surgery, cecal ligation and puncture (CLP) (“septic critical illness”)	(V, H) 24‐week‐old male C57BL/6J mice, human patients with either short or long intensive care unit stay	↑ Bile acids, ↓ FXR and RXR, ↓ basolateral and canalicular transporters, ↑ MRP 3 and MRP 4	17
Vivo: cecal ligation and puncture vitro: +/−medium containing dexamethasone	(V, T) Male Sprague–Dawley rats, hepatocytes derived from rats with sepsis	↓ RXR‐α and FXR; ↓ RXR‐α translocation from cytosol to nucleus, ↓mRNA rBAT (↓ rBAT level); Dexamethasone: reversed sepsis‐inhibited RXR‐α, FXR/RXR binding to rBAT DNA and rBAT protein expression	37
LPS (10 mg/kg i.p) OCA (5 mg/kg gavage)	(V) Male C57BL/6 mice	OCA → ↑ FXR and BSEP, ↓ LPS‐induced hepatocyte apoptosis and inflammatory infiltration, ↓ ALT, AST, TBA and TB,↓ IL‐1β, TNF‐α, IL‐6 → ↓ bile acid synthesis; stimulated ATF4‐mediated autophagy activity	38
NTCP + HNF			
LPS (1 mg/kg body i.p.)	(V) Male Sprague–Dawley rats	↓ HNF 1 and FpB BP → ↓ NTCP mRNA; ↑ NF‐κB and AP‐1	40
LPS (1 mg/kg i.p.)	(V) Male Sprague–Dawley rats (+/− with depletion Kupffer cells)	Complete depletion Kupffer cells:↓ IL‐1β and TNF‐α gene expression, ↓ TNF‐α binding levels, ↑ NTCP RNA, ↓ plasma bile salt, preserved activity of RXR:RAR and HNF1α	39
Activation of LPS/TLR4 signaling pathway			
LPS (1 mg/kg i.p.), LPS (5 mg/kg i.p.)	(V) TLR4‐normal C3H/OuJ mice, TLR4‐mutant C3H/HeJ mice	↓ Oatp4 mRNA levels through TLR4	43
LPS (1 μg/mL) for 18 h	(T) Normal human cholangiocyte	LPS + TLR4 → ↓ ITPR3 via NF‐κB → impairs ductular bicarbonate secretion	23
Activation of PI3K signaling pathway			
Peritoneal contamination and infection	(V) PI3Kγ KO and PI3Kγ KD mice lacking or expressing kinase‐inactive PI3Kγ	↑ PI3K/Akt signaling → ↓ MRP 2 → ↑ hepatic excretory dysfunction	44
Peritoneal contamination and infection with a stool suspension hepatoblastoma cells: a mix of TNF‐α, IL‐1β, IFN‐γ, and LPS	(V, T, H) Male Wistar rats, PI3Kγ−/− (12–16 weeks) mice, human hepatoblastoma cells, plasma from 48 patients fulfilling standard criteria for severe sepsis/septic shock	↑ Plasma bile acids, ↓ BSEP and MRP 2 ↑ PI3K → internalization of pseudovilli	45
Generation of a pro‐inflammatory state			
NO			
NO donors sodium nitroprusside and S‐nitrosocysteine	(T) Human hepatoma cell line stably expressing NTCP (HuH‐NTCP)	NO → S‐nitrosylation of NTCP → ↓ TC uptake ↓ NTCP in the membrane	46
LPS (4 mg/kg body i.p.)	(V) Male Sprague–Dawley rats	↑ Portal and systemic NO_2_^−^ + NO_3_^−^ plasma levels but LPS‐induced NO does not modulate bile formation ↓ HCO_3_^−^, and glutathione output → ↓ bile flow	22
IL‐6			
Cecal ligation and puncture +/‐IL‐6	(V, T) Male Sprague–Dawley rats, IL‐6‐treated cultured hepatocytes from the livers of normal rats	↓ NTCP and MRP 2 transcription → hyperbilirubinemia and cholestasis	47
Media containing IL‐6	(T) Cultured rat hepatocytes	↓ Na + K + ‐ATPase → ↓ sodium‐dependent taurocholate uptake → cholestasis	19
Cecal ligation and puncture +/− recombinant human interleukin‐6	(V) Male adolescent C57Bl6 interleukin‐6 +/+ and interleukin‐6 −/− mice.	Absence of IL‐6 → ↑ hepatic dysfunction and mortality in sepsis ↓ IL‐6 activity → failed bile acid and organic anion clearance, enhanced hepatocellular injury, failed regeneration, poor outcome.	48
IL 8 + CCL2 + CXCL2			
Autologous feces i.p	(V) Domestic female pigs	↑ IL8, CCL2, and CXCL2 → inflammatory reaction and recruitment of monocytes and neutrophils	49
P‐selectin			
A combination of LPS (0.4 mg/kg, i.p.) +/− pretreated with an anti‐P‐selectin antibody	(V) Adult male C57/BL/6 mice	Immunoneutralization of P‐selectin: ↓ leukocyte infiltration, ↓ hepatocellular apoptosis and necrosis, maintains intact bile flow, expression of hepatocyte transporters, and excretory function	50
Mechanisms interfering with aquaporins			
*Salmonella typhimurium* LPS (4 mg/kg body) +/− p75 TNF‐α receptor fusion protein (TNFp75:Fc) (8 mg/kg body wt, 16 h and 1 h before the LPS injection)	(V) Adult male Wistar rats	LPS → ↑TNF‐α → ↓ AQP8 (cytokine‐induced AQP8 proteolysis) → ↓ canalicular membrane water permeability → LPS‐induced cholestasis	51
*S. typhimurium* LPS (4 mg/kg body) +/− adenovectors AdhAQP1	(V) Adult male Wistar rats	AdhAQP1‐treatment improves LPS‐induced cholestasis by stimulating the BSEP/ABCB11‐mediated biliary bile acid excretion	52
Impairment of liver enzymes with hepatic histopathological changes			
*E. coli*‐derived LPS (0.2 mg i.v.), *Staphylococcus aureus* (10^8^ to 10^9^ CFU/mL i.v.), lipoteichoic acid 5 mg i.v.	(H, V) Observational study: patients with *S. aureus* endocarditis and hyperbilirubinemia; New Zealand white rabbits	Hyperbilirubinemia in *S. aureus* sepsis → high risk of dying lipoteichoic acid → defective hepatic excretory function → hyperbilirubinemia	53
LPS (5 mg/kg, i.p.) 100 mg/kg UDCA p.o. for 10 days	(V) Male albino rats	LPS → ↑TBIL, GGT, ALP, AST, ↑ hepatocyte apoptosis, ↑ TNF‐α, IL‐1α, and IL‐4; UDCA →↓ AST, GGT, ALP, ↓ hepatocyte apoptosis, ↓ TNF‐α, ↓ CD3 T‐cell co‐receptor protein, improvement of histopathological features of inflammation	54
	(H) Inpatients who had elevations of ALP above 1000 U/l, observational study	Extremely high elevations of ALP: in sepsis, malignant obstruction, and AIDS	55
Gram‐negative or Gram‐positive infection	(H) Retrospective study, 4 cases with Gram‐negative or Gram‐positive infection	Disproportionately high levels of BT compared to GOT, GPT, LDH, ALP and GGT levels, histological: cholestasis, Kupffer cell hyperplasia and cell infiltration in the sinusoid and portal areas	57
Intraperitoneal sepsis (IS) group by cecal ligation and total parenteral nutrition (TPN) group	(V) Female adult Wistar rats	IS group: degeneration of hepatolobules, enlargement of bile canaliculi with altered microvilli	58
1.75 mL/kg stool suspension i.p.	(V) Male Wistar rats; the organic anionic dyes: indocyanine green and benzopyrylium‐based hemocyanine	Sepsis → liver injury, cholestasis, sinusoidal perfusion impairment → ↓ excretion and accumulation organic anions in the liver parenchyma	59
	(Case report) A 46‐year‐old man with mediastinal abscess that contained acid‐fast bacilli	↑ Conjugated bilirubin, near‐normal ALT, ALP, and PT; after treatment: bilirubin normalization	60
	(Case report) A 58‐year‐old woman septic shock from pneumonia and severe acute respiratory distress syndrome	↑ Bilirubin, GGT and ALP, → sepsis‐related cholestasis	61
	(Case report) A 48‐year‐old woman with bronchopneumonia (*S. aureus*)	Cholestasis and hepatocellular necrosis, hepatosplenomegaly, liver biopsy: intrahepatic cholestasis; after treatment: cholestasis and hepatosplenomegaly disappearance	63
	(Case reports) (1) A 55 year old white female with ulcerative colitis and subphrenic pelvic and lesser sac abscesses. (2) A 66 year old black male with fulminant hepatitis (3) A 58 year old black female with Torulopsis glabrata pneumonia	Intrahepatic cholestasis: inspissated bile within dilated and proliferated portal and periportal bile ductules	64
	(Case report) A 47 years old female with spondylodiscitis and paravertebral abscess	Cholestatic syndrome with jaundice → inflammation‐induced cholestasis	62
Increased mortality			
Patients with different type of sepsis	(H) Observational prospective single‐center study, 608 patients with sepsis	Sepsis‐associated cholestasis was strongly associated with older age, biomarkers of organ dysfunction, and clinical composite scores (APACHE II and SOFA); higher mortality in patients with sepsis‐associated cholestasis	65

↑, 0.029w?>activating, elevation, increase, upregulated; →, results, cause, induce; ↓, reduce, negative regulator, supress, decrease, downregulated, inhibit; AdhAQP1, adenovirus encoding human aquaporin‐1; AIDS, acquired immune deficiency syndrome; ALP, alkaline phosphatase; ALT, alanine aminotransferase; AP‐1, activating protein 1; AQP8, aquaporin‐8; AST, aspartate aminotransferase; BA, bile acids; BSEP, bile salt export pump; ecto‐ATPase, ecto‐adenosinetriphosphatase; CCL2, C‐C motif chemokine ligand 2; CD 3, cluster of differentiation 3; CDCA, chenodeoxycholic acid; CFU, colony forming units; CXCL2, C‐X‐C Motif Chemokine Ligand 2; DCA, deoxycholic acid; DNA, deoxyribonucleic acid; FpB BP, footprint B binding protein; FXR, Farnesoid X Receptor; GGT, gamma‐glutamyl transferase; GOT, glutamic oxaloacetic transaminase; GPT, glutamate‐pyruvate transaminase; HCO_3_, bicarbonate; HNF, hepatocyte nuclear factor; i.p., intraperitoneal; i.v., intravenous; ICKT, Inchin‐ko‐to; IL, interleukin; INR, international normalized ratio; IPV, portal vein; ITPR3, type 3 inositol 1,4,5‐trisphosphate receptor; IVC, inferior vena cava; LDH, lactate dehydrogenase; LPS, lipopolysaccharides; mRNA, messenger ribonucleic acid; MRP, multidrug resistance protein; Na + K + ‐ATPase, Na + K + ‐adenosine triphosphatase; NF‐κB, nuclear factor kappaB; NLRP3, nucleotide‐Binding Domain; Leucine‐Rich Repeat Family; Pyrin Domain‐Containing 3; NO, nitric oxide; NTCP, sodium‐taurocholate co‐transporting polypeptide; OATP, organic anions transporting polypeptides; OCA, obeticholic acid; OSTβ, organic solute transporter beta; p.o., per os; PCI, peritoneal contamination and infection; PI3K, phosphatidylinositol 3‐kinase; PT, prothrombin time; rBAT, rat hepatic bile acid coenzyme A‐amino acid N‐acyltransferase; rlst‐1, complementary DNA encoding human liver‐specific organic anion transporter; RXR, Retinoid X Receptor; RXR:RAR, retinoid X receptor‐retinoid acid receptor; SEB, Staphylococcal enterotoxin B; SIRS, systemic inflammatory response syndrome; TBA, total bile acid; TBIL, total bilirubin; TC, sodium‐taurocholate; TLR4, toll‐like receptor 4; TNF‐α, tumor necrosis factor‐α; UDCA, ursodeoxycholic acid.

### Sepsis‐induced cholestasis

#### Mechanisms interfering with membrane pumps activity

The decrease in bile acids uptake and excretion is the main mechanism engendering cholestasis.^11^ Although sepsis does not alter the synthesis and cytosolic transport of bile acids, it impairs bile acids uptake and secretion by upsetting membrane pumps.^11^ Conjugated bilirubin predominance suggests that bilirubin conjugation is not significantly impacted; indeed, depressed bile acids excretion in the bile ducts is one of the main mechanisms of the sepsis‐associated cholestasis.^12^

Sepsis induces cholestasis by reducing the expression of: (i) NTCP, mediated by tumor necrosis factor‐alpha (TNF‐α) and interleukin 1beta (IL‐1β), with consequent diminished bile salts transport from blood into the hepatocyte^13^;(ii) BSEP and MRP 2 export pumps followed by a decrease in bile flow and in the excretion of bile acids^14^, ^15^; (iii) NTCP in conjunction with MRP 2, which depresses bile flow and biliary excretion of bile salts and glutathione.^15^, ^16^ Consistent therewith, a study conducted in rats showed that Inchin‐ko‐to, a herbal medicine, partially prevents sepsis‐associated cholestasis by increasing MRP 2 protein levels, which improves glutathione excretion and bile flow.^16^ In order to compensate for the sepsis‐induced cholestasis, other mechanisms of ridding the hepatocyte of biliary acids are put into motion, mediated by basolateral exporters MRP 4^15^, ^17^ and MRP 3.^12^, ^17^

It was found that in rat hepatocytes, Na + K + ‐adenosine triphosphatase (Na + K + ‐ATPase) protein is located in the sinusoidal membrane, while ecto‐adenosine triphosphatase (ecto‐ATPase) protein, which is essential for liver cell function, resides in the canalicular membrane.^18^ NTCP is a membrane pump whose function is dependent on the Na + K + ‐ATPase.^4^ Endotoxin, particularly IL‐6, reduces the activity of Na + K + ‐ATPase, which is important for maintaining the electrochemical sodium gradient and implicitly for NTCP activity.^13^, ^19^ Ecto‐ATPase is a bile acid transporter whose level is decreased by lipopolysaccharides (LPS).^20^ Endotoxin and TNF‐α impair the activity of NTCP and canalicular ecto‐ATPase, thereby diminishing taurocholate transport across sinusoidal and canalicular membrane.^20^

An organic anion transporter in the rat liver, rlst‐1, is involved in bile acids and organic anions transport in a sodium‐independent manner. Its downregulation in sepsis reduces bile acids secretion, thus contributing to sepsis‐associated cholestasis.^21^

LPS may induce cholestasis by hindering bile acid‐independent bile flow; it inhibits biliary excretion of glutathione^16^, ^22^ and bicarbonate.^22^, ^23^ Glutathione acts as an osmotic agent important for bile formation.^24^ Altered bicarbonate secretion into the bile may reflect liver impairment.^25^

Sepsis‐associated cholestasis involves: (i) reduced bile acids and organic anion transport^26^ in conjunction with impairment of ATP‐stimulated transport^27^; (ii) decreased bile acids excretion^28^ or decreased basal bile flow and basal bile salt excretion induced by TNF‐α^29^; (iii) reduced bile salts uptake by hepatocyte^30^ with defective bile acids secretion into bile.^31^

#### Mechanisms interfering with nuclear receptors involved in inflammatory responses

**Figure 2:**
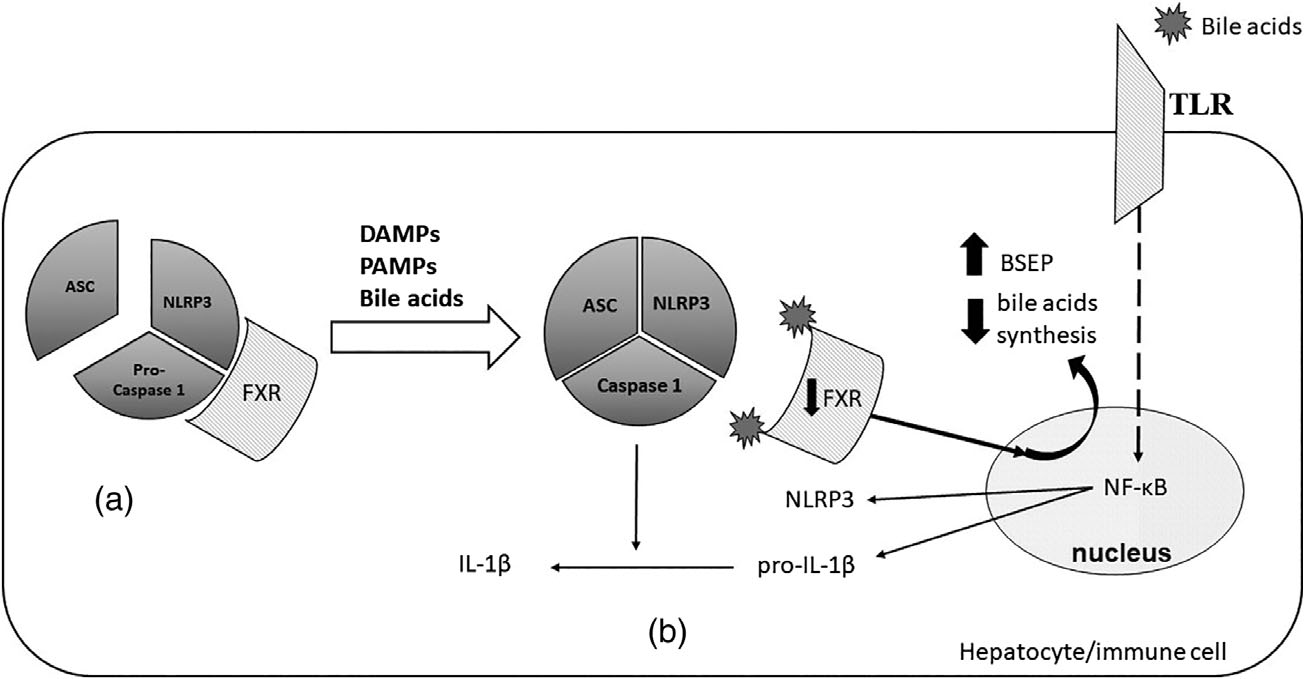
A brief representation of farnesoid X receptor (FXR) role in sepsis‐induced cholestasis. (a) Inflammation‐driven nucleotide‐binding domain (NLRP3) inflammasome consists of intracellular proteins NLRP3, apoptosis‐associated speck‐like protein containing a CARD (ASC), and pro‐caspase 1. FXR, a nuclear receptor, blocks mitochondrial NLRP3 inflammasome assembly by interfering with NLRP3 and pro‐caspase 1. (b) High bile acids levels in the bloodstream lead to: (i) Toll‐like receptor (TLR) ‐nuclear factor‐kappa B (NFκB) pathway activation followed by NLRP3 and pro‐IL‐1β synthesis; (ii) FXR downregulation, which promotes NLRP3 inflammasome assembly with consequent caspase‐1 activation engendering IL‐1β from pro‐IL‐1β. FXR activation by bile acids depresses bile acids synthesis and induces bile salt export pump (BSEP) expression. DAMPs, danger‐associated molecular patterns; IL, Interleukin, leucine‐rich‐containing family, pyrin domain‐containing‐3PAMPs, pathogen‐associated molecular patterns.

The farnesoid X receptor (FXR) is a nuclear receptor expressed in many tissues, but especially in liver, where it is activated by bile acids as a monomer or as a heterodimer in association with retinoid X receptor (RXR).^32^ FXR is involved in bile acid homeostasis, its activation closing a negative feedback loop that limits bile acids synthesis.^33^ FXR induces BSEP expression involved in bile salts excretion from hepatocytes into the bile ducts.^34^ FXR blocks mitochondrial NLRP3 inflammasome assembly by interfering with NLRP3 and caspase 1 (Fig. 2).^35^ NLRP 3 inflammasome consists of intracellular proteins: nucleotide‐binding domain, leucine‐rich repeat family, pyrin domain‐containing 3 (NLRP3), an apoptosis‐associated speck‐like protein (ASC), and pro‐caspase 1 found in innate immune cells, mainly in macrophages.^36^ Inflammasome activation by pathogen‐associated molecular patterns (PAMPs) or danger‐associated molecular patterns (DAMPs) drives a pro‐inflammatory response.^35^ In sepsis‐associated cholestasis, bile acids, acting as DAMPs, generate and maintain inflammation.^35^ LPS (i) downregulate FXR expression in liver cells and macrophages^17^, ^35^, ^37^ and (ii) increase bile acids levels in the bloodstream by suppressing NTCP and BSEP membrane pumps, by upregulating organic solute transporter beta (OSTβ) pump involved in bile acids transport from enterocyte into blood,^35^ and by increasing the expression of basolateral exporters MRP 3 and MRP 4.^17^ Therefore, FXR downregulation leads to NLRP3 inflammasome assembly and activation.^35^ Increased bile acids levels in the bloodstream results in: (i) NLRP3 and pro‐IL‐1β synthesis via toll‐like receptor‐nuclear factor‐kappa B (TLR‐NF‐κB) pathway; (ii) NLRP3 inflammasome assembly with consequent activation of caspase‐1.^36^ (Fig. 2).

Both FXR and RXR may be involved in sepsis‐associated cholestasis as they are suppressed in sepsis.^17^, ^37^ Hepatic bile acid coenzyme A‐amino acid N‐acyltransferase catalyzes an important step in the conjugation of bile acids; its activity is modulated by the heterodimer FXR‐RXR, being suppressed as FXR‐RXR translocation into the nucleus decreases.^37^ A study performed on mice revealed that obeticholic acid, a FXR agonist, improves FXR and BSEP expression and reduces inflammation, but stimulates autophagy in hepatocytes.^38^

The NTCP mRNA expression may be reduced by downregulation of transcriptional activators: hepatocyte nuclear factor (HNF) 1 and retinoid X receptor‐retinoid acid receptor (RXR:RAR)^39^ or HNF 1 and Footprint B binding protein (FpB BP).^40^ It has been shown that depletion of Kupffer cells, which are responsible for cytokine release, leads to the preservation of NTCP expression by maintaining RXR:RAR and HNF 1 activity.^39^

#### Activation of LPS/TLR4 and PI3K signaling pathways

**Figure 3:**
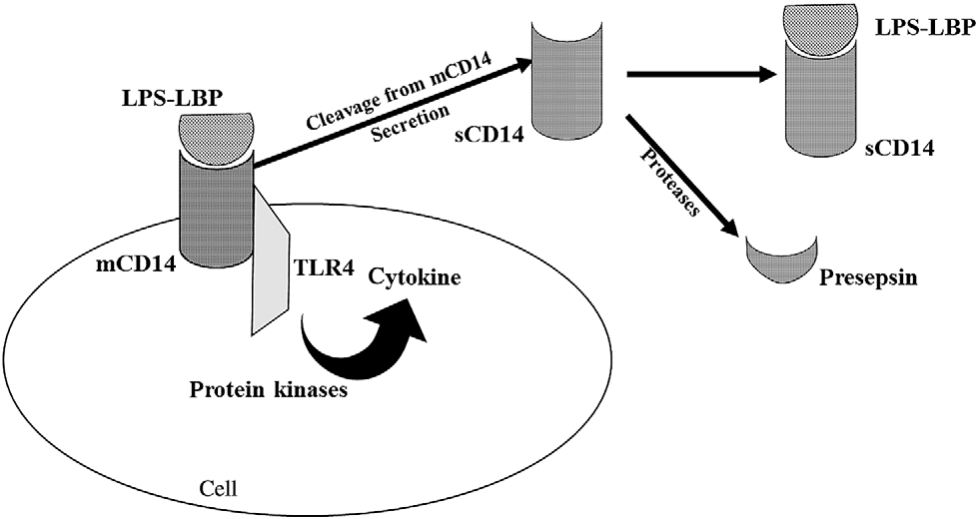
Bacterial lipopolysaccharides binding to membrane‐bound CD14 (mCD14) activates toll‐like receptor 4 (TLR4), thus switching on protein‐kinases and cytokine synthesis. The soluble CD14 (sCD14) in plasma either originates from mCD14 cleavage or is secreted by various cells. Binding of sCD14 to lipopolysaccharides generates a complex that activates various cells, triggering immune responses. Cleavage of sCD14 by proteases produces presepsin. CD14, cluster of differentiation 14; LBP, lipopolysaccharides binding protein; LPS, lipopolysaccharides.

LPS are components of the membrane of gram‐negative bacteria that bind to the cluster of differentiation 14 (CD14) and initiate the systemic inflammatory response.^41^ CD 14 is a glycoprotein receptor attached to the membrane of monocytes, macrophages, and neutrophils, named membrane‐bound CD14 (mCD14).^42^ Through mCD14, LPS activate toll‐like receptor 4 (TLR 4), followed by stimulation of various kinase proteins that spur cytokine production.^42^ Furthermore, TLR 4 activation is followed by a decrease in OATP4 mRNA levels.^43^ Moreover, nuclear factor‐κB (NF‐κB), activated by TLR 4, reduces type 3 inositol trisphosphate receptor (ITPR3) (an intracellular Ca2+ release channel found in cholangiocytes), which impairs bile formation, contributing to cholestasis.^23^ The soluble form of CD 14 (sCD14) found in plasma originates from monocytes, macrophages, and granulocytes either by secretion or by being cleaved from their membrane^42^; sCD14 may bind to LPS and form a complex that activates various cells, thereby driving the immune response; sCD14 may be cleaved by various proteases, thus yielding presepsin, a 13 kDa fragment closely correlated with bacterial infections (Fig. 3).^42^

Phosphatidylinositol‐3‐kinase (PI3K) signaling is involved in liver dysfunction by reducing MRP 2 plasma membrane levels^44^ in conjunction with decreasing BSEP^45^ and by disturbing the cytoskeleton, which results in microvilli effacement and consequent impairment of bile acid and organic anion transport.^45^

#### Generation of pro‐inflammatory state

In sepsis, Kupffer cells release pro‐inflammatory cytokines, reactive oxygen species, and nitric oxide. The interaction of endothelial cells with Kupffer cells leads to an increase in the synthesis of IL 1, IL6, and nitric oxide, the latter interfering with the intrahepatic and systemic circulation.^12^ Nitric oxide promotes sepsis‐associated cholestasis by decreasing NTCP in the plasma membrane and by increasing NTCP S‐nitrosylation followed by inhibition of sodium‐taurocholate uptake.^46^ LPS increases portal and systemic nitric oxide, which does not necessarily affect bile formation.^22^

LPS stimulates sinusoidal endothelial cells, Kupffer cells, and hepatocytes to produce IL‐6, which is central to hepatic inflammatory response.^4^ Moreover, endotoxins induce cytokine production by Kupffer cells, including TNF‐alpha, which in its turn favors IL‐6 synthesis by hepatocytes.^4^ IL‐6 contributes to cholestasis by decreasing NTCP and MRP 2 transcription^47^ and by inhibiting Na + K + ‐ATPase, thus depressing sodium‐dependent taurocholate uptake.^19^ Despite these deleterious actions, a drop in IL‐6 activity is associated with hepatic dysfunction and mortality in sepsis, indicating severe structural hepatic impairment.^48^

The inflammatory response to sepsis leads to: (i) an increase in the concentration of cytokines and chemokines, including IL8, CCL2, and CXCL2, which promote monocytes and neutrophils recruitment^49^; (ii) leukocyte infiltration mediated by P‐selectine,^50^ which lowers bile flow and biliary excretion (hence cholestasis) and induces hepatocellular apoptosis and necrosis.

#### Mechanisms interfering with aquaporins

Another mechanism by which LPS provoke cholestasis relies on aquaporins. By downregulating aquaporin‐8, TNF‐α decreases canalicular membrane water permeability, thus contributing to sepsis‐associated cholestasis.^51^ Furthermore, adenovirus‐mediated transfer of human aquaporin‐1 gene into the liver cells may reduce LPS‐induced cholestasis by improving BSEP activity.^52^

#### Impairment of liver enzymes with hepatic histopathological changes as a response to sepsis

Sepsis may be associated with elevation of liver and biliary enzymes suggesting cholestasis: hyperbilirubinemia due to abnormal hepatic excretory function,^53^ associated with increased alkaline phosphatase (ALP),^54^, ^55^ gamma‐glutamyl transferase (GGT), aspartate aminotransferase (AST), alanine aminotransferase (ALT).^54^ On pathological specimens, bile acids have been noticed both in the bile ducts and in the liver cells cytoplasm. Bile acids can be found in the perisinusoidal spaces and are taken up by the Kupffer cells, leading to their hyperplasia.^11^ Other findings in sepsis‐associated cholestasis include mononuclear cells infiltrating portal space, steatosis, dilated portal, and periportal bile ducts.^56^

Liver histological changes include Kupffer cell hyperplasia, cell infiltration,^57^ and dilated biliary ductules lined by cells topped by distorted microvilli.^58^ Sepsis may damage sinusoidal perfusion, leading to cholestasis with consequent accumulation of organic anions.^59^ Various combinations of changes in liver enzymes and histology are reported to occur in sepsis‐induced cholestasis: (i) high conjugated bilirubin and almost normal ALT, ALP, and prothrombin time^60^; (ii) increase in total bilirubin, GGT, and ALP, with normal ALT and INR^61^; (iii) high total bilirubin and ALP with near‐normal ALT and AST^62^; (iv) liver histological changes and reactive intrahepatic cholestasis.^63^, ^64^

Sepsis‐induced cholestasis may result from the direct action of the bacterial components or from the immune response to infection. The advent of cholestasis is associated with higher mortality in patients with sepsis.^65^ Sepsis‐induced cholestasis seems to correlate with old age, organ dysfunction markers, and APACHE II and SOFA scores.^65^

## Conclusions

Cholestasis and sepsis occurring simultaneously may lie not only in a synchronous relation by originating from a common etiology (such in the case of cholangitis), but also in a diachronic relation, one (sepsis) being the cause of the other (cholestasis); the diachronic relationship is actually more common than the synchronous one and should therefore be the first suspicion when the serum level of biliary enzymes is increased, sometimes accompanied by conjugated hyperbilirubinemia, in a septic patient.

Numerous studies have shown that LPS‐induced sepsis may lead to cholestasis by: (i) interfering with: (a) membrane pumps' activity, (b) aquaporins, and (c) nuclear receptors involved in inflammatory responses; (ii) activating LPS/TLR4 and PI3K signaling pathways; (iii) generating a pro‐inflammatory state. The reality of sepsis‐associated cholestasis is supported by the conclusions of both experimental and human observational studies, and is corroborated by case reports.
